# Supplementary material for: DCDC2 READ1 regulatory element: how temporal processing differences may shape language
Source: Proc Biol Sci. 2020 Jun 3;287(1928):20192712. doi: 10.1098/rspb.2019.2712 (PMC7341942; doi:10.1098/rspb.2019.2712)
Supplement: Supplementary Materials for DCDC2 READ1 regulatory element: how temporal processing differences may shape language [file rspb20192712supp1.pdf]

Supplementary Materials for

DCDC2 READ1 regulatory element: how temporal processing differences may shape language

Kevin Tang[1,†], Mellissa M. C. DeMille[2,†], Jan C. Frijters[3], Jeffrey R. Gruen[2,4,*].

Correspondence to: jeffrey.gruen@yale.edu

Journal: Proceedings of the Royal Society B: Biological Sciences

DOI: 10.1098/rspb

This PDF file includes:

Materials and Methods

Tables S1 to S4

Figures S1 to S3

Materials and Methods

Subjects

Sequence data from 2,138 subjects previously described from 43 populations (DeMille et al., 2018) archived by Dr. Kenneth Kidd and Dr. Judith Kidd (Kidd-Lab), were combined with 1,254 subjects of 13 populations from the 1,000 Genomes Project (Consortium et al., 2015) for a total of 3,392 subjects from 51 populations (SI Table S1).

READ1 genotyping

READ1 alleles were collapsed into three genotypes as previously described (DeMille et al., 2018): RU1-1 has a single copy of repeat unit 1 (RU1); RU1-2 has a tandem duplication of RU1; and a microdeletion of 2,445 bp surrounding READ1. RU1-1 and RU1-2 were extracted from variant call format (vcf) files from the additional 1,254 samples added from 1,000 Genomes Project using indel rs200283737 located in RU1. The microdeletion was genotyped using the esv3608367 structural variant.

Phonological database

Phoneme counts and phonological feature counts were extracted from the Phonetics Information Base and Lexicon (PHOIBLE)(Moran, McCloy, & Wright, 2014) and RUHLEN(Creanza et al., 2015) databases using the Dediu/Moisik toolkit (Dediu & Moisik, 2016, 2015). Priority was given to PHOIBLE if a language existed in both databases. We consulted the literature for the phoneme inventory of three languages, Zaramo (Nurse, 1979; Nurse & Philippson, 1975),

Sardinian (Wikipedia, 2016), and Ancient Hebrew (Rendsburg, 1997), that were missing from both databases and extracted their count data using the Dediu/Moisik toolkit.

Language assignment

Populations were assigned International Organization for Standardization (ISO) 639–3 codes that were used for comprehensive representation of language names, corresponding to the language(s) used by that population (SI Table S1). For populations with multiple languages listed, we used the most ancient language available. Populations were only included if there was adequate historical documentation to support assignment and if there were DNA samples available from more than 20 individuals. Pidgin and creole languages were excluded because they are strongly associated with language contact and borrowing (Bickerton, 1977; McWhorter, 2000). Three populations were excluded because their languages (Bukusu, Hakka and Mende) contain phonemes that could be counted as both a nasal and a stop ("prenasalized" stops).

Merging population samples

We merged Yoruba, Finnish, and Japanese samples from the 1,000 Genomes Project and Kidd-lab collections because they had low F_{st} values and similar READ1 frequencies. Within the Kidd-Lab collection, we combined the two Russian population samples (Vologda and Archangelsk) because they also had a low F_{st} value, similar READ1 frequencies, and shared the same language.

Genetic relatedness

We adjusted for general genetic relatedness (Creanza et al., 2015), by employing Hudson's estimator of F_{st} (Hudson, Slatkin, & Maddison, 1992) as formulated Bhatia 2019 (Bhatia, Patterson, Sankararaman, & Price, 2013; Chaichoompu et al., n.d.) because it is robust to differences in sample sizes between populations. The estimation of pairwise F_{st} values was calculated using 164 informative SNPS (SI Table S2) (Kidd et al., 2011) that accurately capture ancestral relationships between populations. The pairwise F_{st} values between the 51 populations were then organized into a 51-by-51 distance matrix of genetic relatedness. The first three principal components (PCs) of the F_{st} scores were selected using a screeplot (SI Figure S1) and accounted for 98.4% of the genetic variation between populations.

Geographical proximity

To adjust for confounding due to possible language contact, we modeled migratory distances between populations and the putative location of human origin using an undirected acyclic graph with nodes corresponding to the respective 51 populations in our sample. Six hub nodes were also instantiated for five migratory waypoints defined by Ramachandran, et al., (2007) (Ramachandran et al., 2005),

located in Cairo (30N, 31E), Istanbul (41N, 29E), Phnom Penh (12N, 105E), Anadyr (64N, 177E), and Prince Rupert Island (54N, 130W), as well as at one putative location of human origin in South Africa (22S, 20E) proposed by Henn, et al. (2011)(Henn et al., 2011). Edges were also drawn connecting South Africa to Cairo, Cairo to Istanbul, Cairo to Phnom Penh, Cairo to Anadyr, and Anadyr to Prince Rupert Island. Each of the 51 populations were then connected to the hub node where their migration passed through most recently. Each edge was weighted by the great circle distance between the respective two nodes it connected. The pairwise migratory

distances between the 51 populations were then organized into a 51-by-51 distance matrix of geographical proximity. The first three principal components (PCs) of the migratory distances were selected using a screeplot (SI Figure S2) and accounted for 93% of the geographic proximity between populations.

Linguistic relatedness

To adjust for confounding due to possible linguistic relatedness in terms of their sound inventory, we modeled the degree of sound inventory overlap between the languages spoken by the 51 populations. The overlap coefficient was used to quantify the degree of overlap, which we defined as the size of the union of one sound inventory set with a second sound inventory set over the size of the smaller of the two sets. The distance between any two sound inventories was obtained by taking one minus the overlap coefficient. The pairwise sound inventory distances between the 51 populations were then organized into a 51-by-51 distance matrix of linguistic relatedness. The first four principal components (PCs) of the sound inventory distances were selected using a screeplot (SI Figure S3), and accounted for 67.7% of the linguistic relatedness between populations.

Modeling procedure

Generalized linear models were used to predict RU1-1 frequency using the relevant variables that are outlined below as predictors. The models were constructed in *R*, using the *glm* function in the *stats* library. The models were used to investigate the nature of the consonant correlation by examining the different types of consonants as independent variables.

To control for possible confounding, we included the genetic relatedness PCs, the geographical proximity PCs, and the linguistic relatedness PCs as additional fixed effects.

Following standard practice, continuous variables were first log-transformed, then z-score normalized (Baayen, 2008). The PCs were only z-score normalized. The structure of the initial model was:

RU1-1 ~ Stops + Affricates + Fricatives + Nasals + Approximants + Vowels + Tones + Genetic PC-1 + Genetic PC-2 + Genetic PC-3 + Geographical PC-1 + Geographical PC-2 + Geographical PC-3 + Linguistic PC-1 + Linguistic PC-2 + Linguistic PC-3 + Linguistic PC-4

To avoid overfitting, initial models were simplified following a step-down, data-driven selection procedure that compared nested models using the backward best-path algorithm (Barr, Levy, Scheepers, & Tily, 2013). The Akaike information criterion (*AIC)* was used for comparisons. To keep our model maximally conservative, only the linguistic variables (stops, affricates, fricatives, nasals, approximants, vowels, and tones) were considered for exclusion, since the genetic PCs, geographical PCs and the linguistic PCs served as statistical controls. At each step of the selection procedure, models with one linguistic variable excluded were compared, and the model with the lowest AIC was chosen as the best model for the next step. The final best model was obtained when no more linguistic variables could be excluded. The residuals of the best model were found to be normally distributed. No populations were dropped. The structure of the final model was:

RU1-1 ~ Stops + Nasals + Genetic PC-1 + Genetic PC-2 + Genetic PC-3 + Geographical PC-1 + Geographical PC-2 + Geographical PC-3 + Linguistic PC-1 + Linguistic PC-2 + Linguistic PC-3 + Linguistic PC-4

The statistical significance of the individual predictors in the best model was evaluated by 10,000 permutations for each model using the *boot.ci* function in the *boot* library (Canty & Ripley, 2015). Bootstrapped p-values and 95% confidence intervals were computed for each predictor in each model (SI Table S3). To ensure that the results were not driven primarily by a particular set of data points, we applied a leave-k-out method from $k = 1$ to 30 for the best model over the 51 populations (SI Table S4). We estimated the extent to which two predictors are robust, and the minimal number of populations required by our model to observe the effects of stops and nasals, by evaluating a range of k values. For $k = 1$ (also called the jack-knife method), one population was dropped at a time, and therefore 51 models were fitted. For $k = 2$ and $k = 3$, all possible combinations of populations were dropped, with $k = 2$ yielding 2,550 combinations and $k = 3$ yielding 62,475 combinations. Since the number of possible combinations increases non-linearly as k increases, 100,000 combinations were sampled and dropped for each of the higher k's (4 and above). For each k, the fitted models were used to compute the mean, the confidence intervals and the p-value of the coefficients of the stops and the nasals variables. The p-value of each variable was computed as the proportion of models in which the coefficient changed sign. The p-values of the two variables were below 0.05 until $k = 26$ for stops and $k = 23$ for nasals.

Superpop	Population	Popcode	ISO	Language	Macro family	Phoneme source
EUR	Adygei	ady	ady	Adygh	Abkhaz_Adyghe	Ruhlen
AFR	Jew, Ethiopian	etj	ahg	Agaw	Afroasiatic	PH
EAS	Ami	ami	ami	Ami	Austronesian	Ruhlen
MDE	Druze	dru	apc	Eastern Arabic	Afroasiatic	Ruhlen
SAS	Bengali from Bangladesh†	beb	ben	Bengali	Indo_European	SPA
EUR	Chuvash	chv	chv	Chuvash	Turkic	SPA
AMR	Cheyenne	chy	chy	Cheyenne	Algic	Ruhlen
EUR	Danish	dan	dan	Danish	Indo_European	Ruhlen
EUR	Finnish	fin	fin	Finnish	Uralic	SPA
EUR	Irish	iri	gle	Irish Gaelic	Indo_European	SPA
SAS	Gujarati in Houston†	gih	guj	Gujarati	Indo_European	RA
AFR	Hausa	hsa	hau	Hausa	Afroasiatic	SPA
MDE	Jew, Ashkenazi	ash	hbo	Ancient Hebrew	Afroasiatic	suppl
MDE	Jew, Roman	rmj	hbo	Ancient Hebrew	Afroasiatic	suppl
MDE	Jew, Yemenite	ymj	hbo	Ancient Hebrew	Afroasiatic	suppl
EUR	Hungarian	hgr	hun	Hungarian	Uralic	SPA
AFR	Ibo	ibo	ibo	Igbo	Atlantic_Congo	SPA
AFR	Esan in Nigeria†	esn	ish	Esan	Atlantic_Congo	NA
EUR	Toscani in Italia†	tsi	ita	Italian	Indo_European	PH
EAS	Japanese	jpn	jpn	Japanese	Japonic	SPA
SIB	Khanty	kty	kca	Eastern Khanty	Uralic	PH
EAS	Chinese Dai in Xishuangbanna, China†	cdx	khb	Lue	Tai_Kadai	UPSID
EAS	Cambodian	cbd	khm	Cambodian	Afroasiatic	SPA
EAS	Korean	kor	kor	Korean	Koreanic	SPA
SIB	Komi-Zyrian	kmz	kpv	Komi	Uralic	SPA
AMR	Karitiana	kar	ktn	Karitiâna	Tupian	SAPHON

EAS	Laotian	lao	lao	Lao	Tai_Kadai	PH
SAS	Keralite	ker	mal	Malayalam	Dravidian	RA
AFR	Gambian in Western Divisions in the Gambia[†]	gwd	mnk	Mandingo	Mande	PH
EAS	Chinese in Taiwan	cht	nan	Xiamen	Sino_Tibetan	UPSID
EAS	Nasioi Melanesian	nas	nas	Nasioi	South_Bougainville	SPA
EAS	Micronesian	mcr	nau	Nauruan	Austronesian	Ruhlen
AFR	Chagga	cga	old	Kimochi	Atlantic_Congo	GM
AMR	Pima from Arizona, US	pma	ood	Pima	Uto_Aztecan	SPA
SAS	Punjabi from Lahore, Pakistan[†]	pjl	pan	Punjabi	Indo_European	SPA
AMR	Pima from Mexico	pmm	pia	Pima Bajo	Uto_Aztecan	Ruhlen
AMR	Quechua	que	quz	Cuzco-Collao Quechua	Quechuan	SAPHON
EUR	Russian	rus	rus	Russian	Indo_European	SPA
AFR	Sandawe	snd	sad	Sandawe	Isolate	GM
SIB	Yakut	yak	sah	Yakut	Turkic	SPA
EUR	Sardinian	srd	srd	Sardinian	Indo_European	suppl
AMR	Surui	sur	sru	Suruí	Tupian	SAPHON
SAS	Sri Lankan Tamil from the UK[†]	stu	tam	Tamil	Dravidian	PH
EAS	Atayal	atl	tay	Atayal	Austronesian	SPA
AMR	Ticuna	tic	tca	Ticuna	Ticuna_Yuri	SAPHON
SAS	Indian Telugu from the UK[†]	itu	tel	Telugu	Dravidian	RA
EAS	Kinh in Ho Chi Minh City, Vietnam[†]	khv	vie	Vietnamese	Austroasiatic	SPA
AFR	Yoruba	yor	yor	Yoruba	Atlantic_Congo	UPSID
AMR	Maya, Yucatan	may	yua	Yucatec	Mayan	UPSID
EAS	Han	chs	yue	Cantonese	Sino_Tibetan	SPA
AFR	Zaramo	zrm	zaj	Zaramo	Atlantic_Congo	suppl

Popcode	Chrom	RU1M1	RU1_2	Deletion	Cons	Vowels	Tones	Stops	Nasals	Fricatives	Affricates	Approx	DNA source
ady	100	0.130	0.850	0.020	31	2	0	10	2	13	3	3	Kidd
etj	64	0.156	0.844	0.000	28	7	4	12	4	4	4	4	Kidd
ami	76	0.013	0.447	0.539	15	4	0	4	3	4	1	3	Kidd
dru	198	0.071	0.859	0.071	20	6	0	5	2	9	0	4	Kidd
beb	172	0.052	0.773	0.174	36	15	0	16	5	3	4	8	1KG
chv	86	0.023	0.860	0.116	30	9	0	7	5	7	2	9	Kidd
chy	108	0.037	0.826	0.138	11	3	0	4	2	5	0	0	Kidd
dan	98	0.031	0.857	0.112	18	11	0	7	3	6	0	2	Kidd
fin	262	0.024	0.894	0.082	25	16	0	10	5	6	0	4	Kidd+1KG
iri	224	0.036	0.892	0.072	44	20	0	12	9	9	2	12	Kidd
gih	206	0.092	0.772	0.136	36	25	0	16	5	5	4	6	1KG
hsa	76	0.158	0.842	0.000	32	10	3	15	2	7	2	6	Kidd
ash	152	0.072	0.822	0.105	27	8	0	9	2	10	0	6	Kidd
rmj	48	0.085	0.787	0.128	27	8	0	9	2	10	0	6	Kidd
ymj	84	0.155	0.762	0.083	27	8	0	9	2	10	0	6	Kidd
hgr	164	0.037	0.834	0.129	51	15	0	13	6	14	11	7	Kidd
ibo	96	0.198	0.802	0.000	46	16	3	23	6	8	4	5	Kidd
esn	198	0.157	0.843	0.000	25	7	0	8	4	8	1	4	1KG
tsi	214	0.065	0.846	0.089	23	7	0	6	3	5	4	5	1KG
jpn	304	0.040	0.700	0.261	28	11	2	11	3	7	4	3	Kidd+1KG
kty	98	0.020	0.724	0.255	18	13	0	5	4	3	1	5	Kidd
cdx	186	0.005	0.581	0.414	22	9	0	11	3	4	2	2	1KG
cbd	48	0.021	0.625	0.354	21	21	0	9	4	2	2	4	Kidd
kor	106	0.019	0.717	0.264	22	18	0	10	3	3	3	3	Kidd
kmz	94	0.021	0.904	0.074	29	7	0	8	3	9	5	4	Kidd
kar	82	0.000	0.927	0.073	11	20	0	3	4	2	0	2	Kidd

lao	218	0.005	0.679	0.317	28	12	0	15	5	4	0	4	Kidd
ker	52	0.096	0.846	0.058	30	13	0	12	6	5	0	7	Kidd
gwd	226	0.146	0.854	0.000	19	10	5	6	4	3	2	4	1KG
cht	100	0.030	0.760	0.210	19	6	0	10	3	2	4	0	Kidd
nas	46	0.043	0.739	0.217	8	10	0	5	2	0	0	1	Kidd
mcr	74	0.014	0.851	0.135	12	6	0	6	3	0	0	3	Kidd
cga	84	0.131	0.869	0.000	20	5	0	8	3	4	2	3	Kidd
pma	96	0.000	0.874	0.126	19	10	0	8	3	3	2	3	Kidd
pjl	192	0.052	0.786	0.161	50	20	3	24	7	6	6	7	1KG
pmm	192	0.068	0.818	0.115	16	6	0	7	2	3	0	4	Kidd
que	46	0.022	0.739	0.239	26	5	0	12	3	3	3	5	Kidd
rus	162	0.074	0.864	0.062	33	5	0	11	4	11	2	5	Kidd
snd	80	0.225	0.738	0.038	44	15	5	26	2	5	7	4	Kidd
yak	102	0.029	0.647	0.324	33	16	0	9	8	7	4	5	Kidd
srd	60	0.000	0.850	0.150	27	5	0	8	4	7	4	4	Kidd
sur	94	0.000	0.915	0.085	19	10	0	7	4	2	2	4	Kidd
stu	204	0.049	0.799	0.152	21	12	0	8	4	1	2	6	1KG
atl	82	0.000	0.675	0.325	19	8	0	5	3	6	1	4	Kidd
tic	102	0.069	0.578	0.353	15	18	0	8	4	1	0	2	Kidd
itu	204	0.074	0.735	0.191	41	11	0	17	6	6	6	6	1KG
khv	198	0.020	0.586	0.394	21	11	6	7	4	7	0	3	1KG
yor	354	0.167	0.825	0.008	18	11	0	7	2	4	1	4	Kidd+1KG
may	104	0.029	0.837	0.135	20	10	0	8	2	3	4	3	Kidd
chs	102	0.010	0.713	0.277	22	5	5	8	5	3	2	4	Kidd
zrm	66	0.136	0.848	0.015	19	5	0	8	4	4	0	3	Kidd

Table S1.

Populations in study. Included are the world region where the population is located (Superpop : AFR = Africa, MDE = Middle East, EUR = Europe, SAS = South Asia, EAS = East Asia, AMR = America, SIB = Siberia), population name (Population), population

abbreviation (Popcode), language name (Language), abbreviation for the language assignment (ISO), language family (Macro family), database sources for phoneme counts (Phoneme source), the number of chromosomes (Chrom), frequency of READ1 variables (RU1-1, RU1M1 and Deletion), DNA databases used (DNA sources: Kidd denotes the Kidd-lab (DeMille et al., 2018), 1KG denotes the 1,000 Genomes Project (Consortium et al., 2015)), listing of numbers of consonants (Cons), vowels (Vowels), tones (Tones), and the five types of consonants (Stops, Nasals, Fricatives, Affricates, Approximants (Approx)). The symbol † denotes the added populations compared to DeMille et al. (2018) (DeMille et al., 2018).

Chromosome	rsID	Hg19 coordinate
1	rs2986742	6550376
1	rs6541030	12608178
1	rs647325	18170886
1	rs4908343	27931698
1	rs1325502	42360270
1	rs12130799	55663372
1	rs3118378	68849687
1	rs3737576	101709563
1	rs7554936	151122489
1	rs2814778	159174683
1	rs1040404	168159890
1	rs1407434	186149032
1	rs4951629	212786883
1	rs316873	242342504
2	rs798443	7968275
2	rs7421394	14756349
2	rs1876482	17362568
2	rs1834619	17901485
2	rs4666200	29538411
2	rs4670767	37941396
2	rs13400937	79864923
2	rs3827760	109513601
2	rs260690	109579738
2	rs6754311	136707982
2	rs10496971	145769943
2	rs10497191	158667217
2	rs2627037	179606538
2	rs1569175	201021954
3	rs10510228	2208832
3	rs4955316	30415612
3	rs9809104	39146429
3	rs6548616	79399575
3	rs12629908	120522716
3	rs12498138	121459589
3	rs9845457	135914476
3	rs734873	147750355
3	rs2030763	179964727

3	rs1513181	188574996
4	rs9291090	5390637
4	rs4833103	38815502
4	rs10007810	41554364
4	rs385194	85309078
4	rs3811801	100244319
4	rs1693425	100266112
4	rs7657799	105375423
4	rs2702414	179399523
5	rs316598	2364626
5	rs16891982	33951693
5	rs37369	35037115
5	rs6451722	43711378
5	rs12657828	79085726
5	rs6556352	155471714
5	rs1500127	165739982
5	rs7722456	170202984
5	rs6422347	177863083
6	rs1040045	4747159
6	rs2504853	12535111
6	rs7745461	21911616
6	rs2397060	51611470
6	rs192655	90518278
6	rs3823159	136482727
6	rs4463276	145055331
6	rs4458655	163221792
6	rs1871428	168665760
7	rs731257	12669251
7	rs917115	28172586
7	rs32314	32179124
7	rs2330442	42380071
7	rs4717865	73454199
7	rs705308	97695363
7	rs7803075	130742066
7	rs10236187	139447377
7	rs6464211	151873853
8	rs10108270	4190793
8	rs3943253	13359500
8	rs1471939	28941305
8	rs1462906	31896592

8	rs12544346	86424616
8	rs6990312	110602317
8	rs2196051	122124302
8	rs7844723	122908503
8	rs2001907	140241181
8	rs1871534	145639681
9	rs10511828	28628500
9	rs3793451	71659280
9	rs2306040	93641199
9	rs10513300	120130206
9	rs3814134	127267689
9	rs2073821	135933122
10	rs3793791	50841704
10	rs4746136	75300994
10	rs4918664	94921065
10	rs4918842	115316812
10	rs4880436	134650103
11	rs10839880	7850316
11	rs1837606	15838137
11	rs2946788	24010530
11	rs174570	61597212
11	rs11227699	66898492
11	rs948028	120644447
12	rs2416791	11701488
12	rs1513056	17407792
12	rs214678	47676950
12	rs772262	56163734
12	rs2070586	109277720
12	rs2238151	112211833
12	rs671	112241766
13	rs9319336	27624356
13	rs7997709	34847737
13	rs1572018	41715282
13	rs2166624	42579985
13	rs7326934	49070512
13	rs9530435	75993887
13	rs9522149	111827167
14	rs1760921	20818131
14	rs2357442	52607967
14	rs1950993	58238687

14	rs8021730	67886781
14	rs946918	83472868
14	rs200354	99375321
14	rs3784230	105679055
15	rs1800414	28197037
15	rs12913832	28365618
15	rs12439433	36220035
15	rs735480	45152371
15	rs1426654	48426484
15	rs2899826	74734500
15	rs8035124	92105708
16	rs4984913	740466
16	rs4781011	10975311
16	rs2269793	19272908
16	rs818386	65406708
16	rs2966849	85183682
16	rs459920	89730827
17	rs1879488	1401613
17	rs4411548	40658533
17	rs2593595	41056245
17	rs17642714	48726132
17	rs4471745	53568884
17	rs2033111	53788280
17	rs11652805	62987151
17	rs10512572	69512099
17	rs2125345	73782191
18	rs4798812	9420504
18	rs4800105	19651982
18	rs2042762	35277622
18	rs7226659	40488279
18	rs7238445	49781544
18	rs881728	59333108
18	rs3916235	67578931
18	rs4891825	67867663
18	rs874299	75056284
19	rs7251928	4077096
19	rs8113143	33652247
19	rs3745099	52901905
19	rs2532060	55614923
20	rs6104567	10195433

20	rs3907047	54000914
20	rs310644	62159504
21	rs2835370	37885625
22	rs1296819	18076546
22	rs4821004	32366359
22	rs2024566	41697338
22	rs5768007	48207872

Table S2.

SNPs used for generating pairwise genetic distances between populations. rsID: SNP accession number. Hg19 coordinate: homo sapiens (human) genome assembly GRCh37 (hg19) from Genome Reference Consortium.

**variable**	**beta**	**SE**	***t*-value**	**CI_{Lower}**	**CI_{Upper}**	***p*-value**	**Sig.**
(Intercept)	0.063	0.004	17.331	0.055	0.072	0.000	***
Stops	0.011	0.005	2.329	0.002	0.021	0.032	*
Nasals	-0.009	0.005	-1.674	-0.019	0.003	0.080	·
Genetic PC-1	0.001	0.014	0.095	-0.045	0.038	0.897	
Genetic PC-2	-0.062	0.015	-4.026	-0.092	-0.023	0.001	***
Genetic PC-3	0.023	0.010	2.217	-0.002	0.042	0.020	*
Geographic PC-1	0.038	0.015	2.495	-0.004	0.075	0.017	*
Geographic PC-2	-0.004	0.014	-0.308	-0.036	0.040	0.609	
Geographic PC-3	0.016	0.009	1.869	-0.007	0.046	0.270	
Linguistic PC-1	-0.002	0.005	-0.369	-0.013	0.008	0.803	
Linguistic PC-2	-0.004	0.005	-0.841	-0.015	0.007	0.412	
Linguistic PC-3	-0.005	0.005	-1.077	-0.015	0.005	0.294	
Linguistic PC-4	-0.002	0.004	-0.354	-0.011	0.008	0.773	

Table S3.

Results of the best generalized linear model using RU1-1 as the response variable with the number of stops, the number of nasals and PCs of genetic relatedness, geographical proximity and linguistic relatedness as fixed effects. Included the coefficients (beta), standard errors (SE), *t*-values, bootstrap estimation of the lower bounds (CI_{Lower}), the upper bounds (CI_{Upper}), the *p*-values using 10,000 replicates with 95% confidence intervals and the level of statistical significance (sig.): · (nominally significant, p <= 0.1), * (p <= 0.05), ** (p <= 0.01) , *** (p <= 0.001)

k	variable	beta	CI_{Lower}	CI_{Upper}	*p*-value	significance
1	stops	0.011	0.009	0.013	0.000	***
1	nasals	-0.009	-0.011	-0.008	0.000	***
2	stops	0.011	0.009	0.013	0.000	***
2	nasals	-0.009	-0.012	-0.007	0.000	***
3	stops	0.011	0.008	0.014	0.000	***
3	nasals	-0.009	-0.012	-0.006	0.000	***
4	stops	0.011	0.008	0.014	0.000	***
4	nasals	-0.009	-0.013	-0.006	0.000	***
5	stops	0.011	0.008	0.014	0.000	***
5	nasals	-0.009	-0.013	-0.005	0.000	***
6	stops	0.011	0.007	0.015	0.000	***
6	nasals	-0.009	-0.014	-0.005	0.000	***
7	stops	0.011	0.007	0.015	0.000	***
7	nasals	-0.009	-0.014	-0.005	0.000	***
8	stops	0.011	0.007	0.015	0.000	***
8	nasals	-0.009	-0.015	-0.004	0.000	***
9	stops	0.011	0.006	0.016	0.000	***
9	nasals	-0.009	-0.015	-0.004	0.000	***
10	stops	0.011	0.006	0.016	0.000	***
10	nasals	-0.009	-0.015	-0.004	0.001	***
11	stops	0.011	0.005	0.016	0.001	***
11	nasals	-0.009	-0.016	-0.003	0.002	**
12	stops	0.011	0.005	0.017	0.001	***
12	nasals	-0.009	-0.016	-0.003	0.003	**
13	stops	0.011	0.004	0.017	0.001	***
13	nasals	-0.009	-0.017	-0.002	0.004	**
14	stops	0.011	0.004	0.017	0.002	**
14	nasals	-0.009	-0.017	-0.002	0.007	**
15	stops	0.011	0.004	0.018	0.003	**
15	nasals	-0.009	-0.018	-0.002	0.009	**
16	stops	0.011	0.003	0.018	0.004	**
16	nasals	-0.009	-0.018	-0.001	0.012	*
17	stops	0.011	0.003	0.018	0.007	**
17	nasals	-0.009	-0.019	-0.001	0.017	*
18	stops	0.011	0.002	0.019	0.008	**
18	nasals	-0.009	-0.019	0.000	0.021	*
19	stops	0.011	0.002	0.019	0.011	*
19	nasals	-0.009	-0.020	0.000	0.027	*
20	stops	0.011	0.001	0.019	0.015	*
20	nasals	-0.009	-0.021	0.001	0.033	*
21	stops	0.011	0.001	0.020	0.019	*
21	nasals	-0.009	-0.021	0.001	0.041	*
22	stops	0.011	0.000	0.021	0.023	*

22	nasals	-0.010	-0.022	0.002	0.049	*
23	stops	0.011	0.000	0.021	0.029	*
23	nasals	-0.010	-0.023	0.002	0.058	·
24	stops	0.011	-0.001	0.022	0.035	*
24	nasals	-0.010	-0.024	0.003	0.067	·
25	stops	0.011	-0.002	0.022	0.045	*
25	nasals	-0.010	-0.024	0.004	0.078	·
26	stops	0.011	-0.003	0.023	0.053	·
26	nasals	-0.010	-0.025	0.005	0.091	·
27	stops	0.011	-0.004	0.024	0.065	·
27	nasals	-0.010	-0.027	0.006	0.103	
28	stops	0.011	-0.005	0.025	0.076	·
28	nasals	-0.010	-0.028	0.007	0.116	
29	stops	0.011	-0.006	0.026	0.092	·
29	nasals	-0.010	-0.030	0.009	0.133	
30	stops	0.011	-0.008	0.028	0.110	
30	nasals	-0.010	-0.031	0.010	0.148	

Table S4.

Leave-*k*-out analyses. Different number of populations were dropped ranging from 1 to 30. For each variable and at each *k*, the mean beta value, the lower and upper bounds ($CI_{Lower,}$, CI_{Upper}) at 95% confidence, and the *p*-value were computed. The level of statistical significance (significance) are denoted with · (nominally significant, p <= 0.1), * (p <= 0.05), ** (p <= 0.001), and *** (p <= 0.0001))

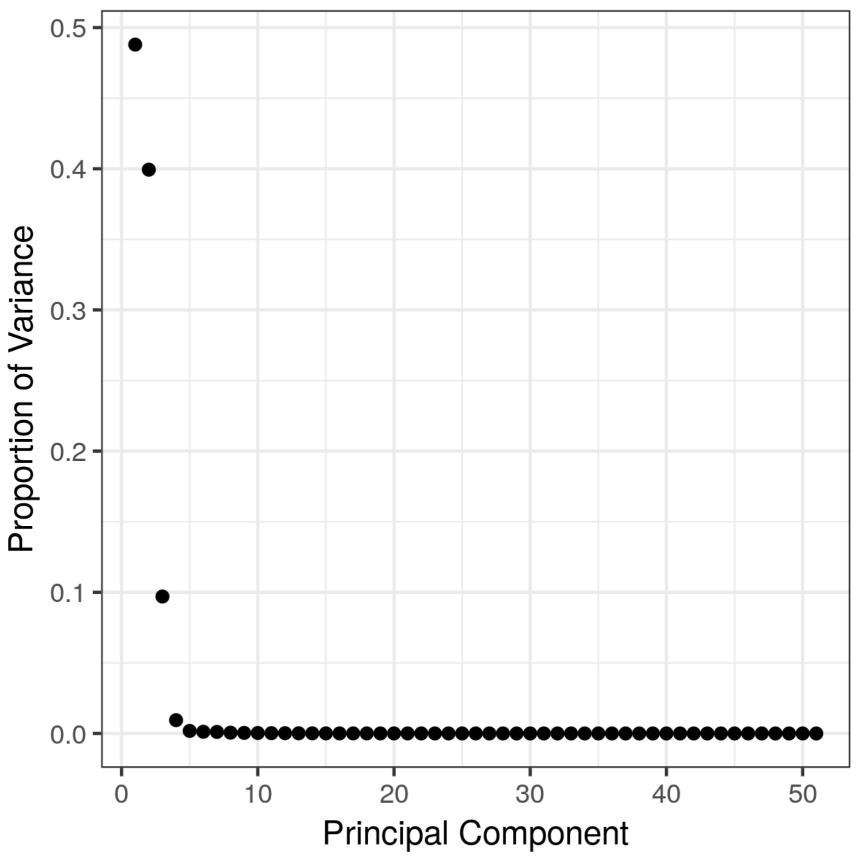

Figure S1.
Distribution of principal component variance of the genetic distance matrix. Principal component one (PC-1, the first point) accounts for 48.79% of the variation, PC-2 (the second point) accounts for 39.94%, and PC-3 (the third point) accounts for 9.70%.

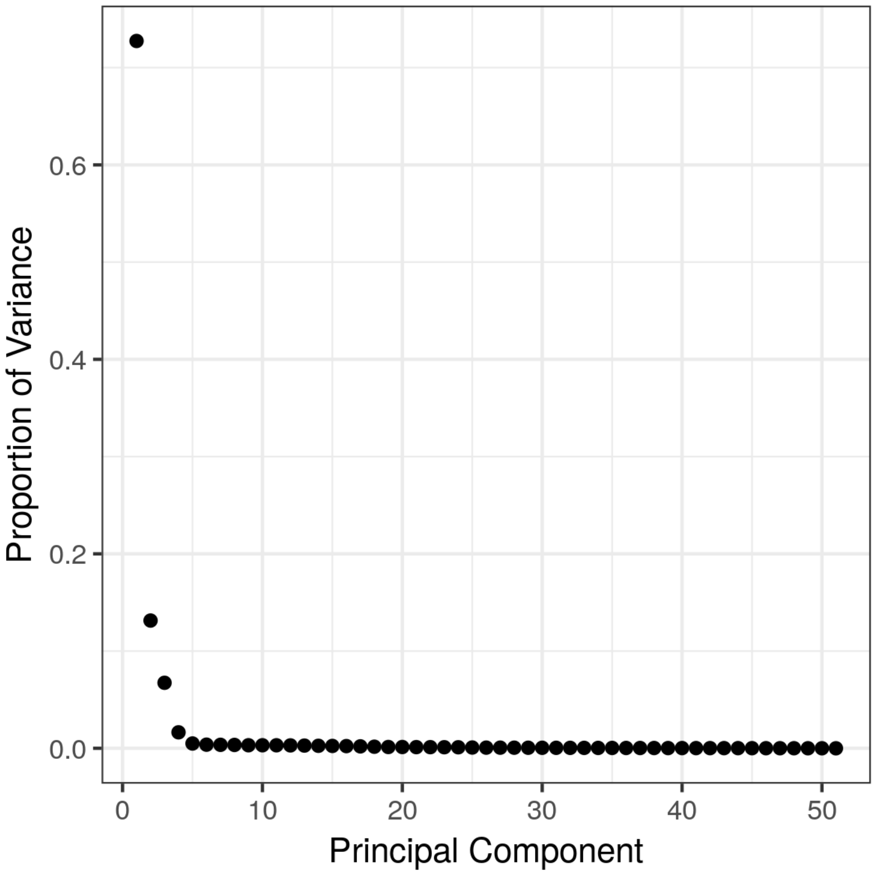

Figure S2.
Distribution of principal component variance of the geographic distance matrix. Principal component one (PC-1, the first point) accounts for 72.74% of the variation, PC-2 (the second point) accounts for 13.14%, and PC-3 (the third point) accounts for 6.73%.

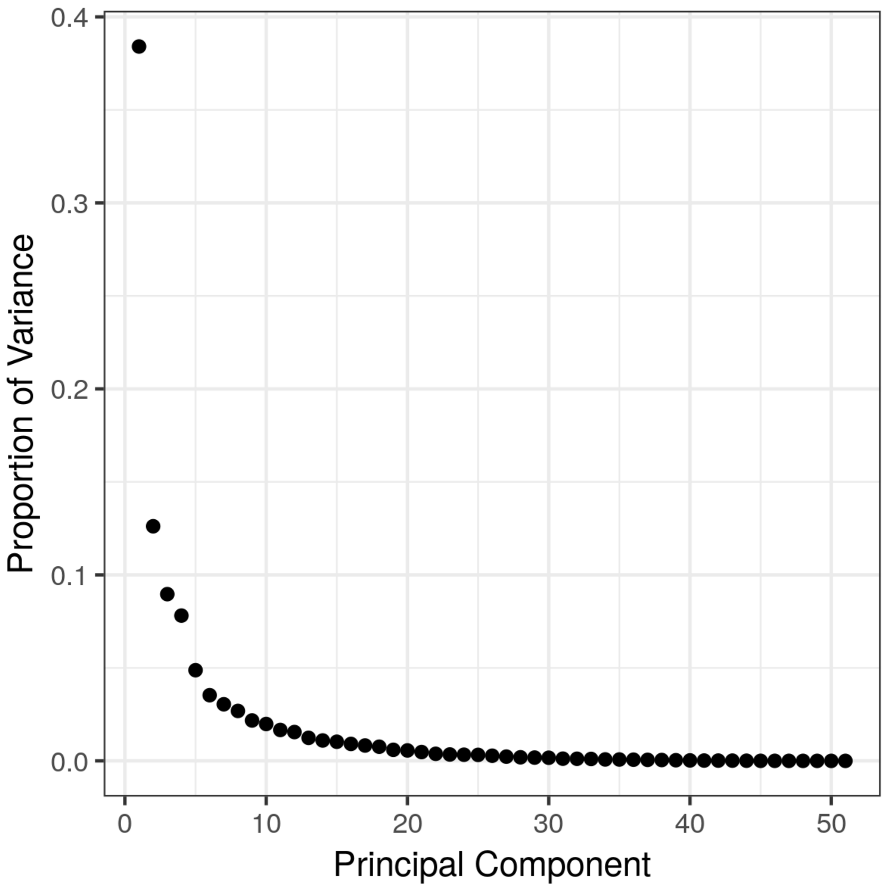

Figure S3.
Distribution of principal component variance of the linguistic distance matrix. Principal component one (PC-1, the first point) accounts for 38.40% of the variation, PC-2 (the second point) accounts for 12.61%, PC-3 (the third point) accounts for 8.96% and PC-4 (the fourth point) accounts for 7.81%.

References

Baayen, R. H. (2008). *Analyzing linguistic data: A practical introduction to statistics using R*. Cambridge, UK: Cambridge University Press. https://doi.org/10.1017/CBO9780511801686

Barr, D. J., Levy, R., Scheepers, C., & Tily, H. J. (2013). Random effects structure for confirmatory hypothesis testing: Keep it maximal. *Journal of Memory and Language*, *68*(3), 255–278. https://doi.org/10.1016/j.jml.2012.11.001

Bhatia, G., Patterson, N., Sankararaman, S., & Price, A. L. (2013). Estimating and interpreting F(ST): The impact of rare variants. *Genome Res*, *23*(9), 1514–1521. https://doi.org/10.1101/gr.154831.113

Bickerton, D. (1977). Pidginization and creolization: Language acquisition and language universals. In A. Valdman (Ed.), *Pidgin and Creole linguistics* (pp. 49–69). Bloomington: Indiana University Press.

Canty, A., & Ripley, B. (2015). boot: Bootstrap R (S-Plus) Functions.

Chaichoompu, K., Abegaz, F., Tongsima, S., Shaw, P. J., Sakuntabhai, A., Pereira, L., & Van Steen, K. (n.d.). KRIS: Keen and Reliable Interface Subroutines for Bioinformatic Analysis. Retrieved September 2, 2019, from https://cran.r-project.org/web/packages/KRIS/KRIS.pdf

Consortium, T. 1000 G. P., Auton, A., Abecasis, G. R., Altshuler (Co-Chair), D. M., Durbin (Co-Chair), R. M., Bentley, D. R., … Marchini, J. L. (2015). A global reference for human genetic variation. *Nature*, *526*, 68 EP-. https://doi.org/10.1038/nature15393

Creanza, N., Ruhlen, M., Pemberton, T. J., Rosenberg, N. A., Feldman, M. W., & Ramachandran, S. (2015). A comparison of worldwide phonemic and genetic variation in human populations. *Proceedings of the National Academy of Sciences*, *112*(5), 1265–1272. https://doi.org/10.1073/pnas.1424033112

Dediu, D., & Moisik, S. (2016). Defining and counting phonological classes in cross-linguistic segment databases. In *LREC 2016: 10th International Conference on Language Resources and Evaluation* (pp. 1955–1962).

Dediu, D., & Moisik, S. R. (2015). Features (and feature counts) from Phoible and Ruhlen's databases [database and software tools]. *GitHub Repository*. GitHub. Retrieved from https://github.com/ddediu/phon-class-counts

DeMille, M. M. C., Tang, K., Mehta, C. M., Geissler, C., Malins, J. G., Powers, N. R., … Gruen, J. R. (2018). Worldwide distribution of the DCDC2 READ1 regulatory element and its relationship with phoneme variation across languages. *Proceedings of the National Academy of Sciences*. https://doi.org/10.1073/pnas.1710472115

Henn, B. M., Gignoux, C. R., Jobin, M., Granka, J. M., MacPherson, J. M., Kidd, J. M., … Feldman, M. W. (2011). Hunter-gatherer genomic diversity suggests a southern African origin for modern humans. *Proceedings of the National Academy of Sciences of the United States of America*, *108*(13), 5154–5162. https://doi.org/10.1073/pnas.1017511108

Hudson, R. R., Slatkin, M., & Maddison, W. P. (1992). Estimation of Levels of Gene Flow from DNA Sequence Data. *Genetics*, *132*(2), 583–589.

Kidd, J. R., Friedlaender, F., Pakstis, A. J., Furtado, M., Fang, R., Wang, X., … Kidd, K. K. (2011). Single nucleotide polymorphisms and haplotypes in Native American populations. *American Journal of Physical Anthropology*, *146*(4), 495–502. https://doi.org/10.1002/ajpa.21560

McWhorter, J. (Ed.). (2000). Language change and language contact in pidgins and creoles (Vol. 21). John Benjamins Publishing, Amsterdam.

Moran, S., McCloy, D., & Wright, R. (Eds.). (2014). *PHOIBLE Online*. Leipzig: Max Planck

Institute for Evolutionary Anthropology. Retrieved from https://phoible.org/
Nurse, D. (1979). Description of 14 Bantu Languages of Tanzania. *African Languages*, *5.1*, 1–150.
Nurse, D., & Philippson, G. (1975). *The Tanzanian language survey.* Dar es Salaam.
Ramachandran, S., Deshpande, O., Roseman, C. C., Rosenberg, N. A., Feldman, M. W., & Cavalli-Sforza, L. L. (2005). Support from the relationship of genetic and geographic in human populations for a serial founder effect originating in Africa. *Proceedings of the National Academy of Sciences of the United States of America*, *102*(44), 15942–15947. https://doi.org/10.1073/pnas.0507611102
Rendsburg, G. A. (1997). Ancient Hebrew Phonology. *Phonologies of Asia and Africa*, *1*, 65–83.
Wikipedia. (2016). Sardinian Language.
